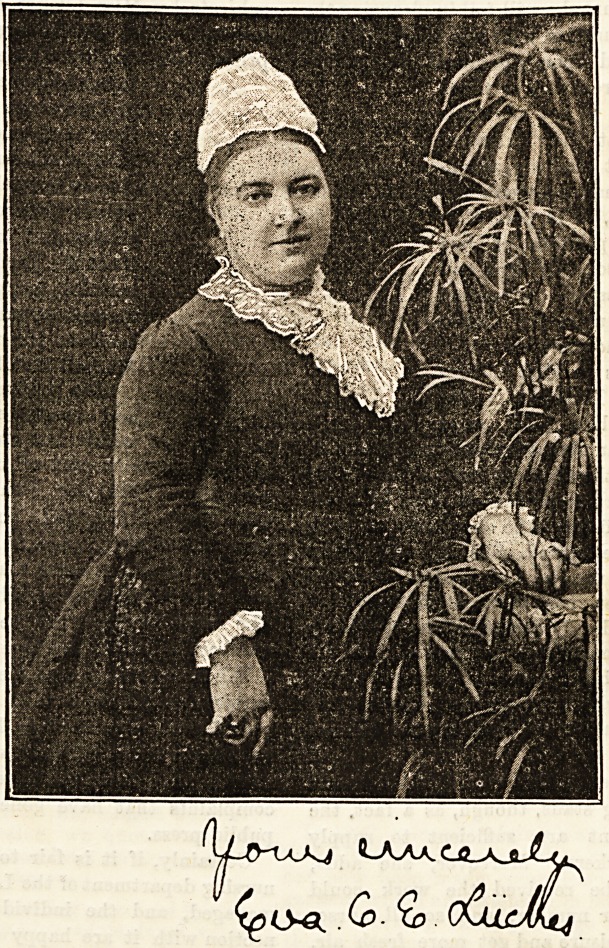# A Celebrated Matron

**Published:** 1890-10-11

**Authors:** 


### A Celebrated Matron.

If on the sudden he begin to rise,
No man that lives can count his enemies!—*Middleton.*

AFTER the recent pictures that have been publicly drawn of a band of martyred nurses, whose wretched lives are spent in a ruthless, "leisureless toil," tending the sick at the London Hospital, by night and by day, without a minute of rest or a sufficiency of food, one is apt to stand aghast at the hard heartedness of the matron, upon whose shoulders the blame of all is laid, and exclaim, with Randolph:—

Hyæna, crocodile, and all beasts of craft,
Have been distilled to make one woman!

Yet Miss Lückes, whose portrait is given below, has the kindest and most pleasing of faces, is courteous and affable, and is decidedly young for the responsible position she holds, and has held during the last ten years at the great hospital in the East of London. Miss Lückes was born July 8th, 1854, and first left her luxurious home in Gloucestershire to take up nursing work purely for the love of being useful in the world, and with a view to returning, after receiving a thorough training, and relieving the poor and the sick in the neighbourhood of her home and parish. She was but twenty-two when she first met the rough experiences that nurses had to contend with in those days, and commenced her probationership. Miss Lückes gained her certificate at Westminster Hospital, and has also had experience at the Hip Disease Hospital for Children, and at the Great Ormond Street Children's Hospital, whilst she was also for a short time night sister at the London Hospital. But some people are fated to rise suddenly, and this was the case with Miss Lückes, who quietly and quickly assumed the position of matron at the Children's Hospital, Pendlebury. It would seem from this that the care of little sufferers was Miss Lückes' favourite branch of nursing, but, at any rate, she soon began to take what must be called a philosophical view of her work, and to interest herself more with the management of nursing staffs, so as to ensure proficiency and success, than with the smaller details of individual attendance on the sick. It was this wide grasp of her professional work that fitted her for the position she was elected to occupy so soon afterwards (September, 1880), and which she has filled ever since, viz., the matronship of the London Hospital.

Yours sincerely
Eva C. E. Lückes

Few do much more than qualify themselves for their future work in the first four years of their training, but Miss Lückes at six-and-twenty stood in a position so proud and responsible that any woman twenty years her senior would have been glad enough to occupy it. And now, though only in the prime of early womanhood, she works with the experience that ten years of constant supervision have given. So much for her early character and her first steps in life.

The only way to further unravel the history and character of this busy and energetic lady, is to trace the history of the growth and development of the nursing department at the London Hospital. Her whole time seems to have been devoted to planning a new system, or improving an old one, and as her life is practically spent within the hospital precincts, and her leisure hours in the apartments specially provided for her in the Nursing Home, her history and that of the institution to which she is so devoted, are closely bound up together.

The standard of nursing in 1880 was very different to what it is now. In its general sphere, as well as in details, a nurse's work required less ability, education, or discernment. For instance, it was quite exceptional for her to take a temperature, and no doctor would delegate the responsibility to her. But now, every nurse must know how to handle a thermometer just as skilfully and well as her sisters of former days were expected to wield the mop and the broom, and to cook their own and the patients' dinners.

One consequence of the increased responsibilities that are now borne by members of the nursing profession, is an alteration in the medical treatment of patients, which goes to show how important a person the silent figure in uniform is becoming, and how much the physician of the future will depend upon her for the performance of a host of services which the patient of a past generation had to do altogether without. But heavy responsibilities are best borne by educated persons, and even the simpler principles of medicine and surgery that nurses are now taught in their lectures, demand a capacity and a degree of intelligence that was altogether foreign to the ancestor of the nurse of 1890. How Miss Lückes came to know all that she does know is a mystery, for though in the days of her training there were few lectures or similar means of acquiring a knowledge of details, she amply proved that she understands every duty that a nurse can be called upon to perform by the publication in 1884 of a book on general nursing (Kegan Paul), and another on the duties of hospital sisters (Churchill), both of which works deal largely and fully, and in the simplest manner, with details.

It is this essentially practical and executive tendency that leads Miss Lückes to hold what she calls—with rather fine sarcasm—the "would-be heroine of the hospital ward" in light esteem. She deprecates hospital work being made the subject of vague ambitions and æsthetic dreams. It is too serious and real, and demands too much labour and self-denying perseverance. So she sends applicants of this description—and they are numerous and easily recognised—quickly about their business, and advises only the more methodical and better ballasted to lead lives in hospital wards.

Though the idea of inaugurating lectures and classes to probationers at the London Hospital was first propounded by Miss Lückes, and although this lady admits the necessity for a high standard of intelligence and education in the wards, she draws the line very distinctly at a point beyond which all this proficiency must not go. The attendant at the sick-bed must never be too intelligent or too highly theoretical to condescend to do a certain amount of menial work. Elevate and develop nursing as you will, there are certain duties that can never be eliminated from it, or entrusted to a less highly trained assistant. "If a nurse cannot, or will not wash up the breakfast things used by her patient," Miss Lückes was once heard to say, "it is possible that she will fail in cleansing the many other articles and instruments with which the patient is brought into contact; and upon the most scrupulous wholesomeness of which his recovery so much depends." In other words, it is not advisable that nurses should forget that their duties, of all others, demand the utmost modesty, attention, and obedience; and that, to reach the greatest excellence in nursing, is to persist in occupying the unobtrusive position of one whose whole personality is absolutely sunk and forgotten, and whose abilities and education and intellect are all centred upon the single, simple desire to minister to another's wants.

Out of the 1,661 applicants to Miss Lückes last year for admission to the London Hospital as probationers, something like 1,500 were disappointed; and as a large number of refusals are met with at other institutions as well, the public may well suppose that the nursing profession must be overcrowded. But not so Miss Lückes, who only turns so many away because her resources are limited. She, in her constant contact with other matrons, nurses, and people connected with hospitals and infirmaries, has been much impressed with what is really an exceedingly curious state of affairs, viz., that whilst there are so many still anxious to join the nursing profession, and though there is so much opportunity for them to exercise it properly, when trained, the number of trained nurses to-day is infinitely too small for the demand that exists for their services.

The reason, says Miss Lückes, is that our hospitals cannot afford to enlarge their nursing staffs, though, as a fact, the patients undergoing treatment are sufficient to supply work for many more workers. Moreover, she adds, if more probationers could be received the work could be distributed over a greater number, and so all nurses would be able to enjoy more leisure and get more fresh air. This, by the way, comes curiously enough from one who is branded as a tyrant whose delight is to grind the nurses down to their "leisureless toil"!

Perhaps the solution of the difficulty may be suggested—though not, indeed, with certainty. If all probationers who present themselves for admission could, in addition to paying for the advantages of instruction and training, maintain themselves entirely without cost to the hospital to which they are attached, then the patients would be attended to by a greater staff, the individual workers would have more time for rest and recreation, whilst, of course, the resources of the institution itself would not be over-taxed. But Miss Lückes always hesitates to express any such opinion as this, or, if she does so, she concludes with the distinct statement that there are many of the most excellent and worthy women who, however deeply inclined to a life's devotion to hospital work, cannot live independently, and, in consequence, if paying probationers alone were received, a very large and valuable section of workers would be excluded from a life to which their inclinations and desires well suit and qualify them.

It may be hoped that the Queen's Jubilee Fund will provide work for a great many more nurses when trained; and Miss Lückes believes there is a wide field in which training might be carried on in workhouses and other similar institutions outside of hospitals.

Though Miss Lückes organised the lectures to nurses at the London Hospital, she always expresses intense gratitude for the assistance rendered her by Dr. Sansom, Physician to the London Hospital, and Mr. Frederick Treves, Surgeon to the London Hospital, who for six and seven years respectively gave up much valuable time to instruct new comers in the theory of nursing. Probationers attend three courses of lectures at the London:—(1) on elementary subjects by Miss Lückes, (2) on physiology, (3) on medical nursing. The courses are followed by an examination, prizes being awarded to the most successful students. The latter have been twice given away by the Duke of Cambridge (who is President of the institution) and once by Professor Huxley. Since 1884, small classes of not more than six probationers in each have been held weekly for individual instruction; and when the lectures and classes and examinations have all been gone through, bandaging is taught, the theoretical training being thus brought to a close. The present lecturers to the probationers of the London Hospital Training School are Mr. Mansell Moullin and Dr. Anderson.

Miss Lückes speaks with the most genuine and unaffected pride of the Nurses' Home, which was erected at the bottom of the great quadrangle at a cost of £10,000. In it each nurse and probationer has her own apartment, besides the use of the large dining-hall and the comfortable and well-furnished sitting-rooms, or rather halls, for recreation. The occupants seem to agree that all has been done to make their existence as happy as possible, and the surroundings as much like home as they can be in an institution where large numbers of people live together.

When the visitor sees these things and, in response to his inquiry, is informed that Miss Lückes planned and completed them all, and when he sees the nurses in their wards with faces that are bright and cheerful he begins to wonder what ground there can have been for all the talk and all the complaints that have gone the round of a portion of the public press.

Certainly, if it is fair to judge from what one sees, the nursing department of the London Hospital is most admirably managed, and the individual nurses who labour in connection with it are happy and contented and loyal to their matron.

We must take farewell of Miss Lückes. Her history, as has been said, is so closely connected with the institution she serves that it has been difficult if not impossible to speak of herself apart from the London Hospital. But both have their interest: the one as a great institution that is performing a noble work, and the other as a woman whose force of character, unceasing energy, and natural ability have raised her among men, and won for her the respect and admiration of all fair-minded persons.

---

Surgeon O'Hara's recommendation of the donkey as a vaccine-lymph agent is not quite practical, for as the donkey is considered by some races to be an unclean animal they would never consent to be saved by its means.

## Figures and Tables

**Figure f1:**